# Supplementary material for: Quantum Confinement Effect in Amorphous In–Ga–Zn–O Heterojunction Channels for Thin-Film Transistors
Source: Materials (Basel). 2020 Apr 20;13(8):1935. doi: 10.3390/ma13081935 (PMC7215306; doi:10.3390/ma13081935)
Supplement: Supplementary file 1 [file materials-13-01935-s001.pdf]

materials

Supplementary Information

Quantum Confinement Effect in Amorphous In–Ga–Zn–O Heterojunction Channels for Thin-Film Transistors

Daichi Koretomo [1,*], Shuhei Hamada [2], Yusaku Magari [1] and Mamoru Furuta [1,2,3]

[1] Engineering Course, Kochi University of Technology, Kami, Kochi 782-8502, Japan; 216007n@gs.kochi-tech.ac.jp (Y.M.); furuta.mamoru@kochi-tech.ac.jp (M.F.)
[2] Material Science and Engineering course, Kochi University of Technology, Kami, Kochi 782-8502, Japan; 225109c@gs.kochi-tech.ac.jp
[3] Center for Nanotechnology, Research Institute, Kochi University of Technology, Kami, Kochi 782-8502, Japan
* Correspondence: 216003c@gs.kochi-tech.ac.jp

The positive bias temperature stress (PBTS) reliability was measured for the homo- and hetero-IGZO TFTs. For the PBTS measurement, a constant V_{GS} of +20 V was applied with the ground for S/D electrodes for 10,000 sec. at 60 °C. Figure S1 shows changes of transfer characteristics of the (a) homo-IGZO-111, (b) homo-IGZO-high-In, and (c) hetero-IGZO (high-In/111 = 10/10 nm) TFTs as function of PBTS time. From the PBTS results, huge ΔV_{th} of +7.0 V was observed from the IGZO-high-In TFT after the PBTS of 10 ks, whereas that of the IGZO-111 TFT was +1.0 V. The V_{th} of both the homo-IGZO TFTs parallel shifted without degradation of subthreshold swing under the PBTS measurement. Generally, the parallel V_{th} shift under PBTS can be explained by a simple charge trapping model caused by interface trap defects [1]. There are some plausible causes for an increase of interface trap defects in the IGZO-high-In TFT. Since the oxygen flow ratio during deposition of the IGZO-high-In layer was substantially higher than that of the IGZO-111 channel for controlling of V_{th}, oxygen ion bombardments would be increased [2,3]. Moreover, a reduction of Ga content in the IGZO channel is also possible cause for the large ΔV_{th} of the IGZO-high-In TFT under the PBTS, because it may induce creation of oxygen vacancies in the IGZO channel, which act as interface trap defects [4,5]. In case of the hetero-IGZO TFT with the high-In/111 = 10/10 nm, transfer characteristics parallel shifted without degradation of subthreshold swing under the PBTS measurement. ΔV_{th} of the hetero-IGZO TFT with the 10/10 nm was +1.2 V after the PBTS of 10,000 s. In addition, ΔV_{th} of the hetero-IGZO TFTs with the 2.5/10 and 5.0/10 nm were +1.3 and +1.2, respectively, whose values are almost the same as that with the 10/10 nm.

Figure S1d shows the relationship between μ_{FE} and ΔV_{th} (PBTS at 60 °C for 10 ks) of the homo- and hetero-IGZO TFTs. The ΔV_{th} of the homo-IGZO-high-In TFT was +7.0 V, whereas that of the hetero-IGZO TFTs were improved to be approximately +1.2 V regardless of the upper channel layer thickness. Thus, the hetero-IGZO TFTs exhibited high μ_{FE} with an improved PBTS reliability by depositing the IGZO-high-In layer on the IGZO-111 channel. Influence of the bottom IGZO-111 layer on the PBTS reliability of the hetero-IGZO TFT was reported on the previous paper [6].

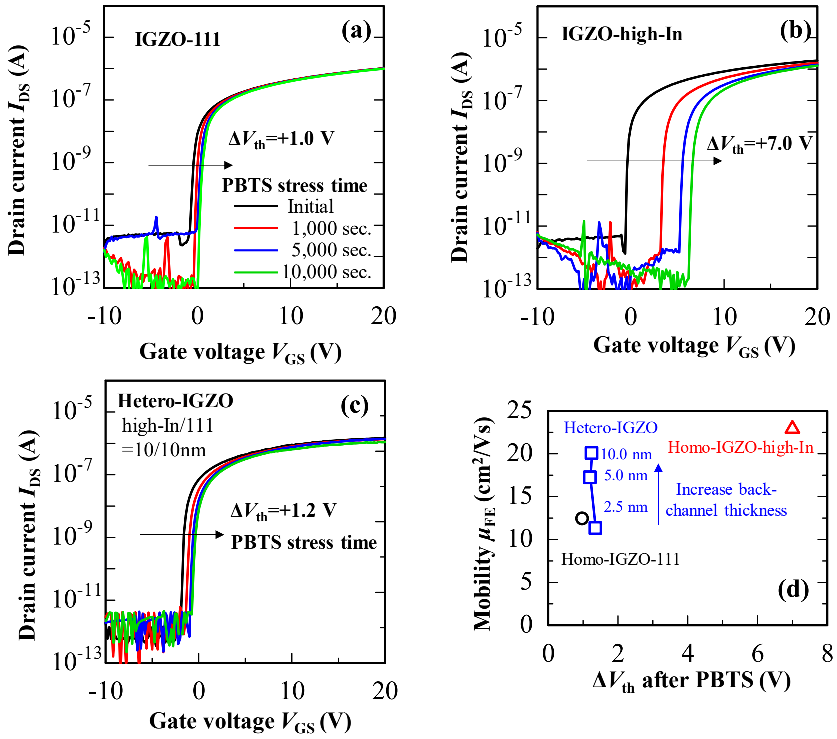

Figure S1. Changes of transfer characteristics of the (**a**) homo-IGZO-111, (**b**) homo-IGZO-high-In, and (**c**) hetero-IGZO TFTs as a function of PBTS time. The thickness of heterojunction channel is IGZO-high-In/IGZO-111 = 10/10 nm. (**d**) Relationship between μ_{FE} and ΔV_{th} after the PBTS of 10,000 s of the homo- and hetero-IGZO TFTs. The stress temperature was 60 °C and stress gate bias was +20 V, respectively.

Figure S2 shows measurement Hall mobilities (experimental results, μ_{Hall}) and mobility model used in the Atlas (simulation, μ_d) for the IGZO-111 and –high-In films as a function of n_e. Hall mobilities were measured by Van der pauw method at room temperature. The calculated mobilities were extracted by Equations (3) and (4) used as simulation parameters as listed in Table 2 in the manuscript. From Figure S2, it was considered that mobility model used in the simulation can be well fitted experimental values of μ_{Hall} for both the IGZO-111 and –high-In films.

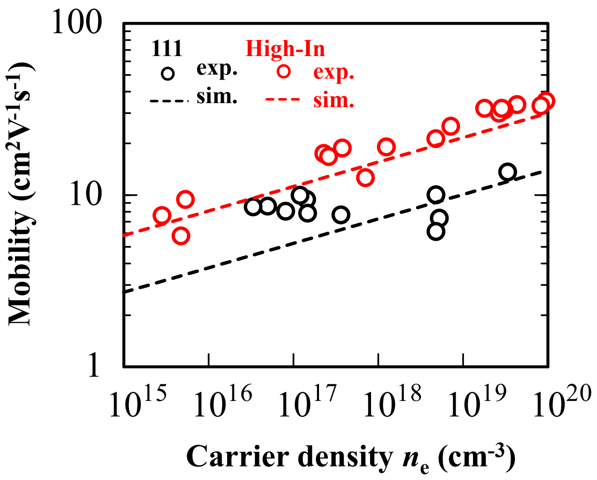

Figure S2. n_e dependences of measured Hall mobilities (circles) and calculated mobilities (lines) of the IGZO-111 and –high-In films.

References

1. Suresh, A.; Muth, J. Bias stress stability of indium gallium zinc oxide channel based transparent thin film transistors. *Appl. Phys. Lett.* **2008**, *92*, 33502, doi:10.1063/1.2824758.
2. Barquinha, P.; Pereira, L.; GonçalvesG.; Martins, R.; Fortunato, E. Toward High-Performance Amorphous GIZO TFTs. *J. Electrochem. Soc.* **2009**, *156*, H161–H168, doi:10.1149/1.3049819.
3. Kim, S.; Jeon, Y.W.; Kim, Y.; Kong, D.; Jung, H.K.; Bae, M.-K.; Lee, J.-H.; Du Ahn, B.; Park, S.Y.; Park, J.-H.; et al. Impact of Oxygen Flow Rate on the Instability Under Positive Bias Stresses in DC-Sputtered Amorphous InGaZnO Thin-Film Transistors. *IEEE Electron Device Lett.* **2011**, *33*, 62–64, doi:10.1109/LED.2011.2173153.
4. Jeong, Y.; Bae, C.; Kim, D.; Song, K.; Woo, K.; Shin, H.; Cao, G.; Moon, J. Bias-Stress-Stable Solution-Processed Oxide Thin Film Transistors. *ACS Appl. Mater. Interfaces* **2010**, *2*, 611–615, doi:10.1021/am900787k.
5. Park, J.H.; Kim, Y.-G.; Yoon, S.; Hong, S.; Kim, H.J. Simple Method to Enhance Positive Bias Stress Stability of In–Ga–Zn–O Thin-Film Transistors Using a Vertically Graded Oxygen-Vacancy Active Layer. *ACS Appl. Mater. Interfaces* **2014**, *6*, 21363–21368, doi:10.1021/am5063212.
6. Furuta, M.; Koretomo, D.; Magari, Y.; Aman, S.G.M.; Higashi, R.; Hamada, S.; Hamada, S. Heterojunction channel engineering to enhance performance and reliability of amorphous In–Ga–Zn–O thin-film transistors. *Jpn. J. Appl. Phys.* **2019**, *58*, 090604, doi:10.7567/1347-4065/ab1f9f.

© 2020 by the authors. Licensee MDPI, Basel, Switzerland. This article is an open access article distributed under the terms and conditions of the Creative Commons Attribution (CC BY) license (http://creativecommons.org/licenses/by/4.0/).
